# Evaluation of drug information resources for drug-ethanol and drug-tobacco interactions

**DOI:** 10.5195/jmla.2019.549

**Published:** 2019-01-01

**Authors:** Robert D. Beckett, Curtis D. Stump, Megan A. Dyer

**Affiliations:** Associate Professor of Pharmacy Practice and Director of the Drug Information Center, College of Pharmacy, Natural & Health Sciences, Manchester University, Fort Wayne, IN, rdbeckett@manchester.edu; Doctor of Pharmacy Student, College of Pharmacy, Natural & Health Sciences, Manchester University, Fort Wayne, IN, cdstump2018@manchester.edu; Doctor of Pharmacy Student, College of Pharmacy, Natural & Health Sciences, Manchester University, Fort Wayne, IN, madyer2018@manchester.edu

## Abstract

**Objective:**

The research evaluated point-of-care drug interaction resources for scope, completeness, and consistency in drug-ethanol and drug-tobacco content.

**Methods:**

In a cross-sectional analysis, 2 independent reviewers extracted data for 108 clinically relevant interactions using 7 drug information resources (Clinical Pharmacology Drug Interaction Report, Facts & Comparisons eAnswers, Lexicomp Interactions, Micromedex Drug Interactions, *Drug Interactions Analysis and Management, Drug Interaction Facts,* and *Stockley’s Drug Interactions*). Scope (presence of an entry), completeness (content describing mechanism, clinical effects, severity, level of certainty, and course of action for each present interaction; up to 1 point per assessed item for a total possible score of 5 points), and consistency (similarity among resources) were evaluated.

**Results:**

Fifty-three drug-ethanol and 55 drug-tobacco interactions were analyzed. Drug-ethanol interaction entries were most commonly present in Lexicomp (84.9%), Clinical Pharmacology (83.0%), and *Stockley’s Drug Interactions* (73.6%), compared to other resources (*p*<0.05). Drug-tobacco interactions were more often covered in Micromedex (56.4%), *Stockley’s Drug Interactions* (56.4%), Drug Interaction Facts (43.6%), and Clinical Pharmacology (41.8%) (*p*<0.001). Overall completeness scores were higher for Lexicomp, Micromedex, *Drug Interaction Facts,* and Facts & Comparisons (median 5/5 points, interquartile range [IQR] 5 to 5, *p*<0.001) for drug-ethanol and for Micromedex (median 5/5 points, IQR 5 to 5, *p*<0.05) for drug-tobacco, compared to other resources. *Drug Interaction Facts* and Micromedex were among the highest scoring resources for both drug-ethanol (73.7%, 68.6%) and drug-tobacco (75.0%, 32.3%) consistency.

**Conclusions:**

Scope and completeness were high for drug-ethanol interactions, but low for drug-tobacco interactions. Consistency was highly variable across both interaction types.

## INTRODUCTION

In the United States, an estimated 88% of the population over the age of 18 reported consuming ethanol at some point in their lives (69% in the past 12 months), and about 51,000,000 adults were current smokers as of 2016 [1, 2]. Ethanol and tobacco have the potential to interact with many prescription and over-the-counter medications that patients may be taking on a daily basis [3, 4]. Drug interactions are a major contributor to adverse drug events, which occur in about 6% of hospitalized patients [5] and are responsible for about 4.5 million outpatient visits each year [6]. Between 33% and 58% of inpatient adverse drug reactions and 42% and 62% of outpatient adverse drug reactions are likely preventable, with drug interactions identified as a key etiology behind adverse drug reactions [7].

Ethanol and tobacco have the potential to interact with therapeutic drugs through several mechanisms [3, 4]. Alcohol dehydrogenase (CYP2E1) is a metabolizing enzyme for ethanol and is inhibited by ethanol; thus, ethanol can interact with substrates, inhibitors, or inducers of CYP2E1 [3]. It can also contribute to additive central nervous system (CNS) depressant effects when used with other CNS depressants (e.g., benzodiazepines, opioids), which can increase a patient’s risk for sedation and psychomotor impairment. Most drug-ethanol interactions are due to the latter mechanism. The large number of compounds found in cigarette smoke induce CYP1A1, 1A2, and 2E1, and suppress CYP2A6, potentially resulting in altered metabolism of drugs affected by these enzymes.

Although there are numerous drug-ethanol and drug-tobacco interactions that may be clinically significant and essential to manage in practice [3, 4, 8, 9], previous evaluations of point-of-care resources have not examined these interaction types [10–13]. The objective of this study was to evaluate point-of-care drug interaction resources for scope, completeness, and consistency in drug-ethanol and drug-tobacco content.

## METHODS

**Figure 1:**
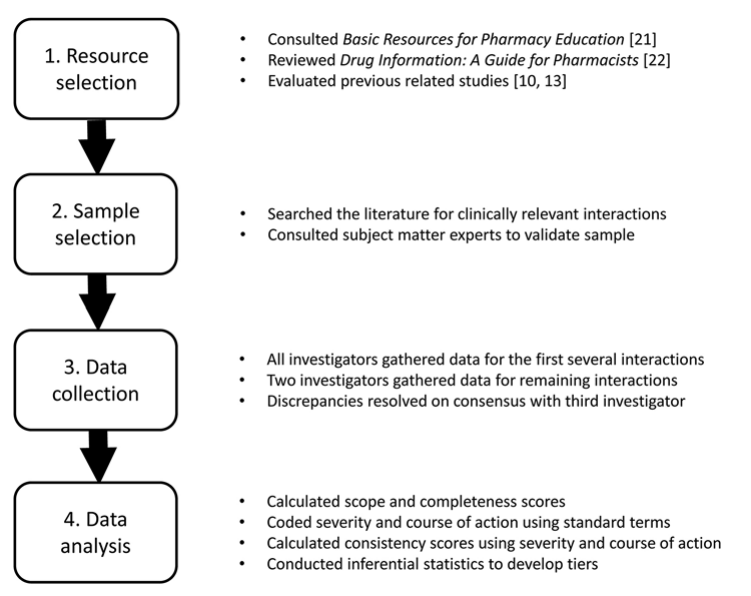
Study methods

This was an observational, cross-sectional analysis of seven drug information resources that pharmacists and other health care professionals commonly use when managing drug interactions. Study methods are outlined in Figure 1. The seven resources included four electronic drug information databases (Clinical Pharmacology Drug Interaction Report [14], Facts & Comparisons eAnswers [15], Lexicomp Interactions [16], and Micromedex Drug Interactions [17]) and three print reference books (*Drug Interactions Analysis and Management* [18], *Drug Interaction Facts* [19], and *Stockley’s Drug Interactions* [20]). The resources were selected through a review of *Basic Resources for Pharmacy Education* [21], published by the American Association of Colleges of Pharmacy (AACP) Library and Information Sciences section (LIS); a seminal book chapter outlining recommended interactions resources that are essential for drug information practice [22]; and a review of similar studies [10, 13].

To compare the seven resources, a sample of drug-ethanol and drug-tobacco interactions was selected. Specific drug-alcohol and drug-tobacco interactions were identified through a review of the Indiana University Department of Medicine Flockhart Table [23] and a PubMed search for systematic reviews describing the most clinically relevant interactions of each type [3, 4, 8, 9]. Drug-tobacco interactions were defined as any interaction between a medication and the components of cigarette smoke, as opposed to specific interactions involving nicotine. The initial list of potential interactions for evaluation was then reviewed by two subject matter experts, a clinical pharmacy specialist in psychiatry and a drug information pharmacist, to ensure all potential interactions were clinically relevant and to identify additional interactions that should be included. None of the evaluated resources were consulted in developing the list to avoid biasing results toward a specific resource.

Several methods were used to promote valid data collection. The reviewers were provided with directions in an orientation session to the project and a data collection form to promote consistency. The data collection form was a shared, cloud-based spreadsheet document that could be updated in real time by multiple users. Data collection for the first several interactions was reviewed by all three investigators to build a common approach. Two independent reviewers then collected data for each of the remaining interactions that were included in the final list. Any discrepancies between the two independent reviewers, thereafter, were resolved by consensus with the third investigator; fewer than ten cases required consensus.

**Figure 2:**
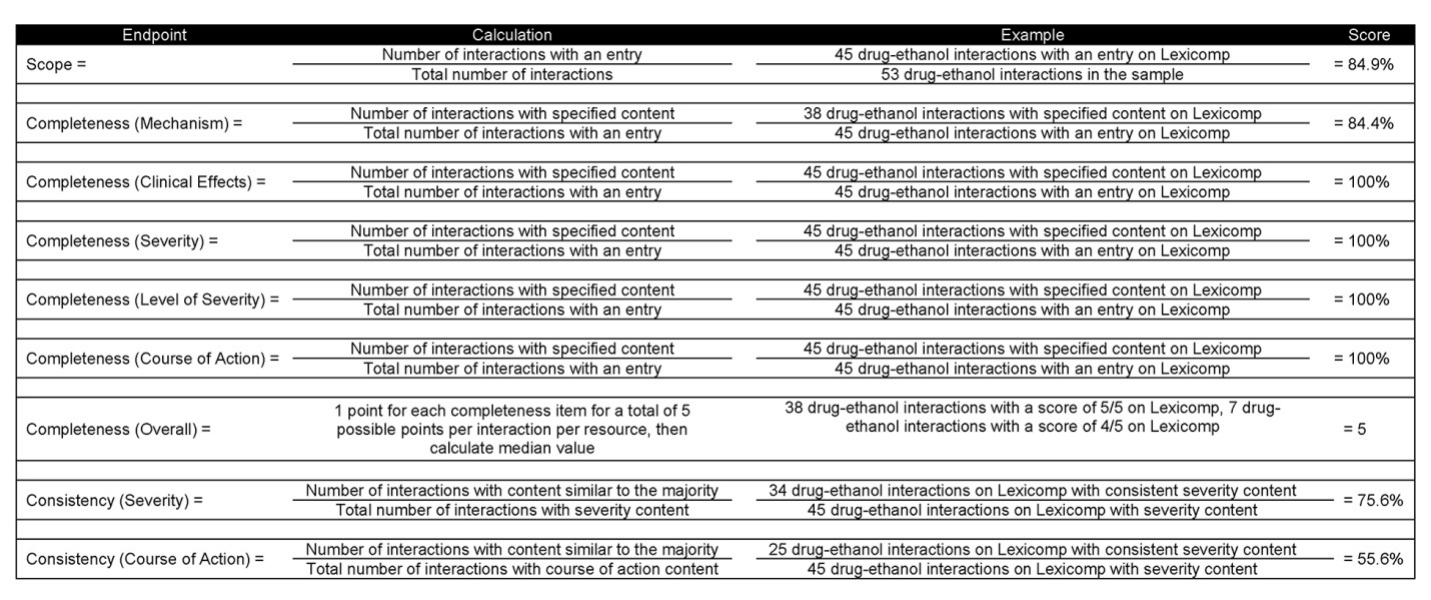
Full description of each endpoint along with an example

Based on previous studies [10–13, 24–27], scope, completeness, and consistency were the evaluated endpoints used to assess the study objectives. Figure 2 provides a full description of how each endpoint was calculated, along with an example. First, scope was defined as presence of an entry and calculated simply as the percentage of sample interactions that had an entry in each resource.

Five items were extracted for each interaction from each resource where an entry was present:

- Mechanism was defined as the proposed pharmacokinetic or pharmacodynamic way the interaction transpires.
- Clinical effects were defined as the potential signs and symptoms resulting from the interaction, as well as patient outcomes.
- Severity was defined as the degree of clinical impact on the patient if the interaction were to occur.
- Level of certainty was defined as the likelihood of the interaction actually occurring, considering how well it is documented in the resource.
- Finally, course of action was defined as the recommended necessary actions if a patient were to present with simultaneous prescriptions or medication orders for the interaction.

Completeness was defined as the percentage of interactions with an entry that provided specified content describing each of the extracted items (i.e., mechanism, clinical effects, severity, level of certainty, course of action). An overall completeness score was calculated for each interaction for each resource with an entry by awarding one point for each of the five assessed items and summing the points. Data were gathered and entered into the data collection form over a three-month period in early 2018. Collected data and definitions align with a previous study [10].

Content for severity and course of action, as the most objective extracted data points, were used as markers to examine consistency among the seven resources. For each interaction, the severity rating for each interaction in each resource was coded as minor, moderate, major, or severe/contraindicated. Consistency scores were calculated as the percentage of severity ratings that were similar to the majority rating from the available sample of resources. A second consistency score was calculated using recommended course of action. Available course of action recommendations were coded as no action needed, monitor, adjust some component of therapy, or avoid the combination, with consistency scores calculated in the same manner as for severity ratings. Resources were omitted from consistency calculations if they had three or fewer entries.

Descriptive statistics, namely, number and percentages for categorical data (i.e., scope scores, completeness items, consistency scores) and median with interquartile range (IQR) for overall completeness scores (given lack of expectation for normal distribution of ordinal data),were primarily used to illustrate results.

To help guide users toward the best resources in terms of scope, completeness, and consistency, we used inferential statistics to group resources into tiers for both drug-ethanol and drug-tobacco content in terms of each of the 3 qualities. The scope, overall completeness, and consistency (for course of action content) scores for each resource were compared to the highest scoring resource in each category. This was done in a stepwise approach guided by similar studies of electronic drug information resources [10, 25]. Resources were grouped into a lower tier when the difference in scores, compared to the highest scoring resource in the next highest tier, was statistically significant, using a 2-sided alpha of 0.05. Resources were omitted from tier calculations if 3 or fewer entries were available for analysis.

Data were treated as paired, since the same interactions were evaluated across the 7 resources; thus, the McNemar test was used to build the scope and consistency tiers (categorical data) and the Wilcoxon signed-rank test was used to build the overall completeness tiers (ordinal data). Inferential statistics were conducted with IBM SPSS Statistics, version 24 [28]. Upon consultation with an institutional review board (IRB) member, it was determined that the study did not involve human subjects and that IRB review was not necessary.

## RESULTS

The initial search identified 37 drug-ethanol and 53 drug-tobacco interactions for analysis. Following review of the initial list by subject matter experts, we added 16 drug-ethanol and 2 drug-tobacco interactions, yielding a total of 108 interactions. The final sample is found in Table 1.

**Table 1:** Sample of interactions for evaluation

Drug-ethanol (n=53)	Drug-tobacco (n=55)
Abacavir	Acetaminophen
Acetaminophen	Alprazolam
Alprazolam	Artemether
Amitriptyline	Caffeine
Aspirin	Carbamazepine
Chlordiazepoxide	Chlordiazepoxide
Chlorzoxazone	Chlorpromazine
Cimetidine	Chlorzoxazone
Clobazam	Clobazam
Clonazepam	Clomipramine
Codeine	Clorazepate
Diazepam	Clozapine
Disulfiram	Codeine
Efavirenz	Cyclobenzaprine
Enflurane	Dexamethasone
Erythromycin	Diazepam
Eszopiclone	Duloxetine
Ezogabine	Enflurane
Felodipine	Estradiol
Fentanyl	Flecainide
Flurazepam	Fluvoxamine
Fluvoxamine	Haloperidol
Fomepizole	Halothane
Gabapentin	Heparin
Halothane	Imipramine
Hydrocodone	Insulin
Hydromorphone	Isoflurane
Isoflurane	Lidocaine
Lorazepam	Melatonin
Maraviroc	Methoxasalen
Metformin	Methoxyflurane
Methadone	Mexiletine
Methamphetamine	Mirtazapine
Methoxyflurane	Nabumetone
Methylphenidate	Naproxen
Metronidazole	Nortriptyline
Midazolam	Olanzapine
Morphine	Ondansetron
Nifedipine	Phenacetin
Oxazepam	Propranolol
Oxycodone	Ramelteon
Pregabalin	Riluzole
Procainamide	Ropinirole
Sevoflurane	Ropivacaine
Sodium oxybate	Selegiline
Temazepam	Sevoflurane
Tetracycline	Tacrine
Tramadol	Theophylline
Triazolam	Tizanidine
Varenicline	Tranylcypromine
Verapamil	Triamterene
Zaleplon	Verapamil
Zolpidem	Warfarin
	Zileuton
	Zolmitriptan

Scope score results are described in Table 2. Of the total sample of 53 drug-ethanol interactions, scope score ranged from 20.8% (*Drug Interactions Analysis and Management*) to 84.9% (Lexicomp). Of the total sample of 55 drug-tobacco interactions, scope score ranged from 10.9% (Facts & Comparisons) to 56.4% (Micromedex, *Stockley’s Drug Interactions*). No drug-tobacco interactions were found in Lexicomp, and we confirmed with a company representative that Lexicomp does not index drug-tobacco interactions (although they did index 3 drug-nicotine interactions at the time of writing).

**Table 2:** Scope scores

Resource	Drug-ethanol (n=53)		Drug-tobacco (n=55)	

	n	%	n	%
CP	44	83.0%	23	41.8%
FC	37	69.8%	6	10.9%
LC	45	84.9%	0	—
MM	35	66.0%	31	56.4%
DIAM	11	20.8%	12	21.8%
DIF	21	39.6%	24	43.6%
SDI	39	73.6%	31	56.4%

CP: Clinical Pharmacology; FC: Facts & Comparisons; LC: Lexicomp; MM: Micromedex; DIAM: *Drug Interactions Analysis and Management;* DIF*: Drug Interaction Facts;* SDI: *Stockley’s Drug Interactions.*

Completeness results are described in Table 3. For drug-ethanol interactions, information was most often provided for clinical effects (ranging from 86.4%, Clinical Pharmacology, to 100.0%, Facts & Comparisons, Lexicomp, Micromedex, *Drug Interactions Analysis and Management*) and course of action (ranging from 76.9%, *Stockley’s Drug Interactions*, to 100.0%, Facts & Comparisons, Lexicomp, Micromedex, *Drug Interactions Analysis and Management*). Clinical effects (ranging from 13.0%, Clinical Pharmacology, to 100.0%, Micromedex and *Drug Interactions Analysis and Management*) and course of action (ranging from 32.0%, *Drug Interaction Facts*, to 100.0%, Facts & Comparisons, Micromedex, *Drug Interactions Analysis and Management*) were also the highest scoring items for drug-tobacco interactions.

**Table 3:** Completeness results for interactions with entries

Resource	Mechanism		Clinical effects		Severity		Level of certainty		Course of action		Overall completeness	

	n	%	n	%	n	%	n	%	n	%	median	IQR
Drug-ethanol interactions												
CP (n=44)	41	93.2%	38	86.4%	44	100.0%	1	2.3%	38	86.4%	4	3.75 to 4
FC (n=37)	34	91.9%	37	100.0%	37	100.0%	37	100.0%	37	100.0%	5	5 to 5
LC (n=45)	38	84.4%	45	100.0%	45	100.0%	45	100.0%	45	100.0%	5	5 to 5
MM (n=35)	31	88.6%	35	100.0%	35	100.0%	35	100.0%	35	100.0%	5	5 to 5
DIAM (n=11)	10	90.9%	11	100.0%	1	9.1%	2	18.2%	11	100.0%	3	3 to 3.5
DIF (n=21)	19	90.5%	19	90.5%	21	100.0%	21	100.0%	19	90.5%	5	5 to 5
SDI (n=39)	32	82.1%	38	97.4%	2	5.1%	8	20.5%	30	76.9%	3	2 to 3
Drug-tobacco interactions												
CP (n=23)	21	91.3%	3	13.0%	23	100.0%	0	—	15	65.2%	3	2 to 3
FC (n=6)	0	—	5	83.3%	6	100.0%	0	—	6	100.0%	3	3 to 3
MM (n=31)	31	100.0%	31	100.0%	31	100.0%	31	100.0%	31	100.0%	5	5 to 5
DIAM (n=12)	11	91.7%	12	100.0%	1	8.3%	3	25.0%	12	100.0%	3	3 to 3.25
DIF (n=25)	21	84.0%	24	96.0%	0	—	3	12.0%	8	32.0%	2	2 to 3
SDI (n=31)	27	87.1%	30	96.8%	2	6.5%	11	35.5%	24	77.4%	3	3 to 3

IQR: Interquartile range; CP: Clinical Pharmacology; FC: Facts & Comparisons; LC: Lexicomp; MM: Micromedex; DIAM: *Drug Interactions Analysis and Management;* DIF*: Drug Interaction Facts;* SDI: *Stockley’s Drug Interactions.*

Level of certainty was the lowest scoring item for both drug-ethanol interactions (ranging from 2.3%, Clinical Pharmacology, to 100.0%, Facts & Comparisons, Lexicomp, Micromedex, *Drug Interaction Facts*) and drug-tobacco interactions (ranging from 0, Clinical Pharmacology, Facts & Comparisons, to 100.0%, Micromedex).

Overall completeness scores ranged from a median of 3 (IQR 2 to 3, *Stockley’s Drug Interactions*) to 5 (IQR 5 to 5, Facts & Comparisons, Lexicomp, Micromedex, *Drug Interaction Facts*) for drug-ethanol interactions and 2 (IQR 2 to 3, Clinical Pharmacology, *Drug Interaction Facts*) to 5 (IQR 5 to 5, Micromedex) for drug-tobacco interactions.

Consistency of drug-ethanol interaction information, when assessed using severity ratings, was highest with Lexicomp (75.6%), followed by *Drug Interaction Facts* (71.4%), Micromedex (68.6%), Facts & Comparisons (51.4%), and Clinical Pharmacology (36.4%). However, when assessed using course of action, it was highest with Micromedex (82.9%), followed by Clinical Pharmacology (81.6%), *Drug Interaction Facts* (73.7%), Facts & Comparisons (56.8%), *Stockley’s Drug Interactions* (56.7%), and Lexicomp (55.6%). For drug-tobacco interactions, consistency scores could only be reported for Micromedex (35.5%) and Clinical Pharmacology (30.4%) for severity. When assessed using course of action, drug-tobacco consistency scores were highest for *Drug Interaction Facts* (75.0%), followed by *Stockley’s Drug Interactions* (50.0%),* Drug Interaction Analysis and Management* (41.7%), and Micromedex (32.3%).

Results of the tier analysis are described in Table 4. Depending on the category, resources were grouped into two to four tiers, based on scores for scope, overall completeness, and consistency. For drug-ethanol interactions, Lexicomp placed in the highest tier for both scope and completeness; whereas for drug-tobacco interactions, Micromedex placed in the highest tier for both scope and completeness. For consistency (in terms of course of action, which had more available data points), Micromedex and *Drug Interaction Facts* placed in the highest tier for both interaction types.

**Table 4:** Tier analysis

	Drug-ethanol			Drug-tobacco		

	Scope	Completeness	Consistency	Scope	Completeness	Consistency
Tier 1	LC, CP, SDI	LC, MM, DIF, FC	MM, CP, DIF	MM, SDI, DIF, CP	MM	DIF, SDI, DIAM, MM
Tier 2	FC*, MM*	CP†, DIAM†	FC*, SDI‡, LC*	DIAM†, FC†	DIAM‡, SDI†, FC*, CP†	N/A
Tier 3	DIF†	SDI†	N/A	N/A	DIF*	N/A
Tier 4	DIAM*	N/A	N/A	N/A	N/A	N/A

CP: Clinical Pharmacology; FC: Facts & Comparisons; LC: Lexicomp; MM: Micromedex; DIAM: *Drug Interactions Analysis and Management;* DIF*: Drug Interaction Facts;* SDI: *Stockley’s Drug Interactions.*

* *p*<0.05 compared to next highest tier,

† *p*<0.001 compared to next highest tier,

‡ *p*<0.01 compared to next highest tier.

## DISCUSSION

This evaluation identified that clinically relevant drug-ethanol interactions were most commonly present in Lexicomp, Clinical Pharmacology, and *Stockley’s Drug Interactions*, whereas drug-tobacco interactions were more commonly identified in the latter two resources as well as Micromedex and *Drug Interaction Facts*. Lexicomp, Micromedex, *Drug Interaction Facts*, and Facts & Comparisons provided the most complete information regarding drug-ethanol interactions, but Micromedex alone provided complete drug-tobacco information. It should be noted that drug-ethanol interaction information scored higher for nearly all resources in terms of scope (except for *Drug Interaction Facts*) and completeness (except for Micromedex and *Stockley’s Drug Interactions*), when compared to drug-tobacco information. Consistency among resources was highly variable, with Micromedex and *Drug Interaction Facts* providing good consistency for both interaction types. There were no substantial differences in scores for electronic versus print resources, when taken as a whole.

Notably, there was no single resource that scored in the highest tier across scope, completeness, and consistency for both interaction types, emphasizing the need for using multiple resources on interactions in practice settings, where pharmacists and other health care professionals rely on such resources at the point of care, as well as in library collections that serve health care professions and educational institutions. Each resource had distinct strengths and limitations depending on interaction type and information of interest, and results from this study can help direct information professionals and users to the higher quality resources depending on specific needs. For example, Micromedex stood out for having the best coverage of drug-tobacco interactions, complete information across assessed items (e.g., mechanism, clinical effects) for both interaction types, and among the highest consistency scores. However, Clinical Pharmacology, Lexicomp, and *Stockley’s Drug Interactions* covered more drug-ethanol interactions.

We also noted that Facts & Comparisons, Lexicomp, and *Drug Interaction Facts* had complete analysis of drug-ethanol interactions, similar to Micromedex. Despite having among the strongest scope and completeness scores for drug-ethanol interactions, Lexicomp notably did not address drug-tobacco interactions.

Differences among resources (especially for *Drug Interactions Analysis and Management* and *Stockley’s Drug Interactions*), in terms of completeness, tended to be driven by deficiencies in severity and level of certainty information. *Drug Interaction Facts*, in particular, only addressed drug-tobacco interactions in a single appendix table, which could contribute to lack of completeness in this area. In addition to helping guide information professionals and users, these results can aid in focusing educational instruction on appropriate use of these resources and help guide collection management during times of budget constraints.

Results of this study echo a previous investigation focused on drug-drug and drug–dietary supplement interactions in several key ways [10]. First, the previous investigation also identified that deficiencies in severity and level of certainty were the most common factors that drove differences in completeness. Both studies identified that the information is highly variable among resources with consistency scores ranging from about 32% to 83% in this study and from about 35% to 70% in the previous study. However, consistency and scope have improved compared to an earlier study [13]. Finally, *Drug Interaction Facts*, Facts & Comparisons, Lexicomp, and Micromedex had the highest completeness scores in 2 past studies [10, 11], which was similar to this study for drug-ethanol interactions, but not drug-tobacco interactions. One key difference was that this study yielded lower scope scores (range of about 21% to 85% for drug-ethanol and 0 to 56% for drug-tobacco) compared to previous investigations (about 67% to 97% and about 71% to 88%), suggesting that, particularly for drug-tobacco, clinically relevant interactions are not sufficiently addressed.

Strengths of this study included use of two independent data collectors with, anecdotally, few discrepancies that needed resolution, investigation into an important category of drug interactions that have not been addressed in previous studies [10–13], and use of seven highly regarded electronic and print point-of-care resources that are recommended by experts [21, 22].

There were also several important limitations. First, although our sample represented a cross-section of the most clinically relevant drug-ethanol and drug-tobacco interactions, not every potential interaction was evaluated. However, the sample size compared favorably to past studies [10–12]. Our sample size was potentially smaller than ideal for analyzing drug-tobacco completeness and consistency, primarily due to lower than expected scope scores. Additionally, we noted that data collection was simpler for resources such as *Drug Interaction Facts,* where information was provided in discrete, easily identifiable categories, compared to others, such as *Stockley’s Drug Interactions*, where information was generally provided in narrative paragraphs. This could potentially lead to artificial differences in completeness scores.

Some previous studies have identified that it would be of interest to evaluate whether resources recommend specific alternate therapies [26, 27]; we did not evaluate alternatives, but this may be an appropriate topic for future investigation. Since we conducted the study using a cross-sectional design, we only analyzed each interaction at a single point in time and did not account for potential longitudinal changes. We attempted to maximize fair treatment of resources by evaluating an interaction in each of the seven resources on the same day. Finally, we noted that it had been several years since each of the three print resources had been updated. Impact of this limitation is expected to be minimal given the lack of newer drugs in our sample.

Future evaluations of resources for analyzing drug interactions should focus on other types of drug interactions with non-drug agents, such as food and illicit substances, that have not been previously examined. Additionally, content describing multidimensional interactions (i.e., interactions that are themselves altered by a third entity) have been minimally evaluated and warrant further study.
